# Subacute Infective Endocarditis of Aortic Valve During Pregnancy

**DOI:** 10.7759/cureus.2748

**Published:** 2018-06-05

**Authors:** Muhammad Wajih Ullah, Sunita Lakhani, Sunder Sham, Fariha Ashraf, Wardah Siddiq, Tariq Siddiqui

**Affiliations:** 1 Cardiology, Mayo Clinic, Rochester, USA; 2 Internal Medicine, Liaquat University of Medical and Health Sciences Hospital Jamshoro Sindh Pakistan., Jamshoro, PAK; 3 Internal Medicine, Ghulam Muhammad Mahar Medical College, Sukkur, PAK; 4 Cardiology, VA Palo Alto Health Care System, Palo Alto, USA; 5 Internal Medicine, Beth Israel Deaconess Medical Center/ Harvard Medical College, Boston, USA; 6 Internal Medicine, Maharashtra Institute of Medical Education & Research, Talegaon, IND

**Keywords:** infective endocarditis, pregnancy complications, bicuspid aortic valve

## Abstract

Bacterial infective endocarditis is a rare but a life-threatening infection during pregnancy. Infective endocarditis during pregnancy is often associated with a congenital heart condition or an earlier history of rheumatic heart disease. In pregnant women with infective endocarditis, the maternal and fetal mortality rate can reach as high as 33% and 29%, respectively. In most cases, infective endocarditis runs a subacute course and involves the mitral valve, nonetheless, rarely it can involve the aortic valve as well. We are documenting a rare case of subacute infective endocarditis in a 26-year-old pregnant female with severe aortic stenosis with associated multiple systemic emboli. The patient was managed by urgent cesarean section at 35 weeks of gestation followed by aortic valve replacement; there was no maternal or fetal mortality. This case report highlights the importance of early diagnosis, and timely management of infective endocarditis in pregnant women to prevent maternal and fetal death.

## Introduction

Bacterial infective endocarditis is a serious life-threatening infection during pregnancy and runs a subacute course. The reported incidence of infective endocarditis during pregnancy is 0.006% and the maternal mortality rate due to the same condition is 33% [1-2]. Further, heart failure or embolic event causes a considerable number of deaths in those patients [3]. A delay in the management of pregnant women with infective endocarditis increases the fetal mortality rate to 29% [4]. In this paper, we present a case of subacute infective endocarditis in a 26-year-old pregnant female with the presenting complaints of fever and shortness of breath for four weeks.

## Case presentation

A 26-year-old pregnant patient was referred from the outpatient Gynecology department to the outpatient Cardiology department with shortness of breath and fever for four weeks. Her shortness of breath was gradual in onset and progressive in nature. The patient reported having shortness of breath after walking three blocks during the early course of the disease. However, she reported having shortness of breath at rest for the past four days. She experienced no associated symptoms such as chest pain, cough, runny nose, rash or any antecedent infection. Patient’s past medical, surgical and family history was unremarkable, and she had no modifiable or non-modifiable cardiovascular risk factors. She had no known allergic reaction to food or drugs. She never smoked cigarettes or used any illicit drugs.

Obstetric and Gynecological history revealed she was gravida 1, para 0, at 35 weeks age of gestation. She had no symptoms until four weeks ago when she suddenly developed fever and shortness of breath.

The patient's vital signs on examination were (1) Temperature: 100.4 F with no associated chills or rigors, (2) Blood Pressure: 110/72 mm Hg, (3) Respiratory Rate: 30 breaths/min, (4) Heart Rate: 102 beats/min. Cardiac examination revealed regular pulse with no radio-radial or radio-femoral delay. On auscultation, an end systolic murmur (3/6 grade) radiating to the carotids at the second and third intercostal spaces was heard. On abdominal examination, the spleen was palpable 1 cm below the subcostal margin. Examination of the soles revealed erythematous lesions near the third and the fourth digits. Rest of the systemic examination was unexceptional.

The patient was admitted to the ward for additional investigations. The initial electrocardiogram (EKG) on admission showed sinus tachycardia without specific ST and T-wave changes. Chest X-ray was insignificant with no signs of pulmonary congestion. Laboratory findings revealed erythrocyte sedimentation rate (ESR) = 102 mm/hr (normal range: 0-29 mm/hr for women), hemoglobin = 8.2 mg/dl (normal range: 12-15.5 mg/dl in women), white blood cells = 13,600/cumm (normal range: 4000-11,000/cumm), platelets = 324,200/cumm (normal range: 150,000-450,000/cumm), serum sodium = 135 mEq/L (normal range: 137-145 mEq/L), serum potassium = 3.6 mEq/L (normal range: 3.5-5.0 mEq/L ), d-Dimers = 390 ng/ml (normal range <500 ng/ml), creatinine = 0.7 mg/dl (normal range: 0.1-1.2 mg/dl). Urine dipstick was negative for proteins and blood. Viral markers for hepatitis B and C were non-reactive. Antinuclear antibody (ANA) titers were in normal range.

Transthoracic echocardiography (TTE) revealed thickened bicuspid aortic valves with restricted movements. Transesophageal echocardiography (TEE) confirmed the findings. The echocardiography showed large vegetations (9 x 11 mm in dimensions) attached to the aortic cusps. Ejection fraction was within the normal range. Two sets of blood cultures were positive for *Staphylococcus Aureus* and displayed sensitivity towards ampicillin, amoxicillin, gentamicin, and clindamycin. Based on clinical signs and symptoms, TTE and TEE findings, and positive blood cultures the patient was diagnosed with infective endocarditis secondary to severe aortic stenosis.

The patient was started on appropriate medications for infective endocarditis (ampicillin + sulbactam 12 gm). Follow-up visit revealed the patient’s condition to be deteriorating despite being compliant with the prescribed antibiotics. After consultation with the obstetrician and the cardiac surgeon, a decision was made to perform an urgent cesarean section followed by aortic valve replacement. Transvaginal ultrasound showed an appropriate for gestational age fetus in vertex presentation. A healthy baby was delivered via cesarean section with no maternal or fetal complications. The patient underwent successful aortic valve replacement three days after her delivery.

The patient was discharged after a five-day observation period. At the fourth-month follow-up visit both the patient and baby were in good clinical health.

## Discussion

Infective endocarditis is a rare but life-threatening infection during pregnancy. Predisposing factors that are associated with infective endocarditis include congenital heart condition, IV drug abuse, periodontal disease, previous history of rheumatic heart disease, and local infection [5]. Over the past few decades, advancement in the medical and surgical management of pregnant women with heart diseases has comparatively reduced the maternal and fetal mortality rates, however, the percentage is still high at 33% and 29%, respectively [3-4].

Infective endocarditis presents with atypical findings in pregnant women and its diagnosis can be challenging for the physicians [6]. When a patient presents with the classical triad of fever, anemia, and heart murmurs, infective endocarditis should be listed as one of the differentials. Fever is seen on every occasion in infective endocarditis. The heart murmurs and anemia can be seen in normal pregnant patients due to physiological effects of pregnancy on the cardiovascular system and blood volume respectively [7]. Thus, pregnant women with an unexplained fever and a cardiac murmur or a pre-existing heart condition should be monitored and investigated further. Early diagnosis and timely management are important in reducing the maternal and fetal mortality rates. TTE, TEE, and blood cultures are important in diagnosing infective endocarditis in pregnant women.

The treatment of choice for active infective endocarditis is antibiotics. Valve replacement is delayed until the infection has been eradicated due to the risk of re-infection. Early clinical management of pregnant women with an active infective endocarditis can reduce the maternal and fetal risk. A review article of 68 cases suggested emergent delivery without regard to measures of fetal lung maturity due to high fetal mortality rates in pregnant women with infective endocarditis [8]. The mode of delivery (vaginal or cesarean section) should be considered individually according to the length of the pregnancy and the condition of the mother. If the condition of the mother is better and cardiac surgery is electively planned after a few days, then antibiotic therapy should be administered, and cesarean section or vaginal delivery is performed [9]. If urgent or emergent cardiac surgery is indicated, cesarean section is usually performed prior to valve replacement surgery [10].

Our patient had infective endocarditis secondary to the fibrosed aortic valve, which is very rare but lethal to both the mother and fetus [11]. The patient, in this case, had the emergent cesarean section followed by successful replacement of aortic valve which helped reduce the risk of mortality in both the mother and the fetus.

## Conclusions

Bacterial infective endocarditis is a serious condition in pregnancy. Patients should be managed by emergent delivery followed by surgical replacement of the valve. Based on our observations, we conclude that early diagnosis based on clinical recognition, echocardiography findings, and appropriate timely management can be lifesaving both for mother and fetus.
